# Increase in salivary oxytocin and decrease in salivary cortisol after listening to relaxing slow-tempo and exciting fast-tempo music

**DOI:** 10.1371/journal.pone.0189075

**Published:** 2017-12-06

**Authors:** Yuuki Ooishi, Hideo Mukai, Ken Watanabe, Suguru Kawato, Makio Kashino

**Affiliations:** 1 NTT Communication Science Laboratories, NTT Corporation, Morinosato Wakamiya Atsugi, Kanagawa, Japan; 2 Department of Computer Science, School of Science and Technology, Meiji University, Tama, Kawasaki, Kanagawa, Japan; 3 Department of Biophysics and Life Sciences, Graduate School of Arts and Sciences, University of Tokyo, Meguro, Tokyo, Japan; 4 Department of Information Processing, Interdisciplinary Graduate School of Science and Engineering, Tokyo Institute of Technology, Midori-ku, Yokohama, Kanagawa, Japan; 5 Core Research for Evolutional Science and Technology, Japan Science and Technology Agency (CREST, JST), Atsugi, Kanagawa, Japan; Universitat Wien, AUSTRIA

## Abstract

Relaxation and excitation are components of the effects of music listening. The tempo of music is often considered a critical factor when determining these effects: listening to slow-tempo and fast-tempo music elicits relaxation and excitation, respectively. However, the chemical bases that underlie these relaxation and excitation effects remain unclear. Since parasympathetic and sympathetic nerve activities are facilitated by oxytocin and glucocorticoid, respectively, we hypothesized that listening to relaxing slow-tempo and exciting fast-tempo music is accompanied by increases in the oxytocin and cortisol levels, respectively. We evaluated the change in the salivary oxytocin and cortisol levels of participants listening to slow-tempo and fast-tempo music sequences. We measured the heart rate (HR) and calculated the heart rate variability (HRV) to evaluate the strength of autonomic nerve activity. After listening to a music sequence, the participants rated their arousal and valence levels. We found that both the salivary oxytocin concentration and the high frequency component of the HRV (HF) increased and the HR decreased when a slow-tempo music sequence was presented. The salivary cortisol level decreased and the low frequency of the HRV (LF) to HF ratio (LF/HF) increased when a fast-tempo music sequence was presented. The ratio of the change in the oxytocin level was correlated with the change in HF, LF/HF and HR, whereas that in the cortisol level did not show any correlation with indices of autonomic nerve activity. There was no correlation between the change in oxytocin level and self-reported emotions, while the change in cortisol level correlated with the arousal level. These findings suggest that listening to slow-tempo and fast-tempo music is accompanied by an increase in the oxytocin level and a decrease in the cortisol level, respectively, and imply that such music listening-related changes in oxytocin and cortisol are involved in physiological relaxation and emotional excitation, respectively.

## Introduction

Music is an art common to all humankind that transcends national and cultural boundaries. Listening to music has a wide range of effects on human beings in that it can excite or relax, and these effects have been widely investigated in brain imaging studies, endocrinological studies and physiological studies involving, for example, the measurement of autonomic responses [1,2]. Listening to exciting music elicits physiological arousal such as an increase in heart rate (HR) [3]. Listening to music often induces intense emotion accompanied by chills and increases in HR and the skin conductance response [4–6]. Listening to music can also have an anti-stress or relaxing effect. The secretion of salivary cortisol induced by a psychological stress task is attenuated by listening to relaxing music [7], and this is more pronounced when the music is in a major key than when it is in a minor key [8]. Listening to relaxing music suppresses stress-induced increases in HR and blood pressure [9]. Listening to music has a wide range of positive effects as noted above, and this has led to the frequent use of music therapy [10,11].

Although listening to music can have a wide variety of effects on human beings such as causing changes in cardiovascular activity and inducing emotion [12–14], the key factors behind these effects have not been fully understood. Tempo has been considered a factor when determining whether the effect of listening to music is exciting or relaxing. Earlier studies demonstrated that listening to music with a fast tempo induces an increase in sympathetic nerve activity [15]. On the other hand, listening to slow tempo music reduces the HR and is felt to be relaxing [16]. The effect of tempo on excitation and relaxation can extend to human behavior and emotions. When walking in synchrony with music stimuli, the walking speed is entrained with a change in the musical tempo if the music is relaxing or activating [17].

However, the chemical bases of the effects of musical tempo on relaxation and excitation remain unknown. Oxytocin is a candidate for the chemical basis of relaxation because oxytocin has anti-stress [18] and anti-anxiety effects [19] as does listening to music. Oxytocin has the potential to facilitate vagal activity [20] and also plays a role in protecting the heart from sympathetic reactivity to stress [21], which implies that an increase in the oxytocin level contributes to the relaxation effect through activation of the vagal nerve. It is reasonable to hypothesize that the sedative effect of listening to music is based on these functions of oxytocin.

It can be assumed that cortisol is a candidate chemical basis of the excitation effect of music listening. Since corticosterone, a glucocorticoid in rodents, increases the firing rate of cardiovascular neurons in the rostral ventrolateral medulla (RVLM), which is the primary regulator of the sympathetic nerve that controls cardiac activity [22]) in rats [23], it is possible that an increase in the cortisol level will contribute to excitation through the activation of the sympathetic nerve. Therefore, it is fair to hypothesize that excitation induced by listening to music is based on the cortisol function.

Taken together, we can expect that listening to relaxing music with a slow tempo would increase the oxytocin level, while listening to exciting music with a fast tempo would increase the cortisol level. The present study compared the oxytocin and cortisol levels in saliva before and after listening to music with fast and slow tempi. In contrast to the collection of blood samples, obtaining saliva samples is non-invasive and the samples include protein-free cortisol that precisely reflects physiological actions that cannot be confirmed based on the total blood hormone levels [24,25]. Carter et al. indicated that salivary oxytocin is a good biomarker with which to monitor central oxytocin function [26].

## Materials and methods

### Ethics statement

Before the experiment, participants were provided with an information sheet that outlined the general purpose of the study and informed them that they could withdraw at any time without penalty. All participants except the author signed the consent form. All methods employed in this study were approved by the Ethics and Safety Committees of NTT Communication Science Laboratories, and were in accordance with the Declaration of Helsinki.

### Participants

Twenty-six healthy males aged 21–34 years (29.4 ± 0.81; mean ± SEM) participated in the experiments. Females were not selected to avoid any effects of the menstrual cycle. All of the participants had normal hearing ability, no specific musical training, and no habit of listening to classic music or piano pieces. The participants were informed that they might hear some pieces of music. They were asked to provide two saliva samples, and to describe their arousal and valence levels, and their familiarity with the pieces of music they heard after the experiment. They gave their informed consent, which was approved by the Ethics Committee of NTT Communication Science Laboratories, and were paid for their participation. The experiments were performed in a sound-insulated room. The participants sat on a sofa. The experiments were conducted between 14:00 and 18:00 h to minimize the effect of circadian rhythms.

### Chemicals

Protease inhibitor cocktail tablets were purchased from Roche Diagnostics (Basel, Switzerland). [3H]-labeled oxytocin was obtained from NEN (USA). Oxytocin ELISA kits were obtained from Enzo Life Sciences (USA). Trifluoroacetic acid (TFA) and acetonitrile (ACN) were purchased from Wako Pure Chemicals (Japan). Other reagents were of the highest commercially available grade.

### Music stimuli

We prepared two contrasting stimuli, namely slow-tempo (149 touches, 56 crotchets/min average) and fast-tempo (417 touches, 233 crotchets/min average) music sequences, both of which lasted 20 min and consisted of several piano pieces composed by Chopin. Here, one touch means striking one note or chord on the piano with the right hand. All the pieces were in a major key and taken from commercially available CDs. The sampling rate and quantization level for all the piano pieces were 44.1 kHz and 16 bits, respectively. To examine the properties of the piano pieces we selected, the following six characteristics were rated by six music experts; tempo (0-slow to 15-fast), rhythm (0-vague to 15-outstanding), pitch level (0-low to 15-high), pitch range (0-narrow to 15-wide), harmonic complexity (0-simple to 15-complex), and consonance (0-dissonant to 15-consonant) [27]. The six music experts consisted of a professional pianist, a semi-professional jazz pianist, a signal-processing researcher who uses music as a sample signal, two semi-professional drummers, and a semi-professional violinist. For the assessment, we prepared a paper on which we had drawn six 15-cm lines [28]. The experts were instructed to make marks somewhere along the lines to rate the features of the music. The names of the piano pieces, their properties and the data of rated music features are shown in S1 Table in Supporting Information. Only the rating score for tempo showed a significant difference between slow- and fast-tempo music sequences (S2 Table). The sound pressure levels (SPL) of all the piano pieces were adjusted so that they did not exceed an A-weighted SPL of 75 dB in the slow mode. The music stimuli were converted to analogue signals with an audio interface (EDIROL UA-5, Roland, Japan) and presented through loudspeakers (CM9, Bowers & Wilkins, UK).

### Self-reported familiarity with music pieces

The participants reported their familiarity with the pieces of music used in the experiment. For the assessment, we prepared a paper on which we had drawn one 15-cm line as well as the music rating features. After all the experiments (after rating arousal and valence on the second day), the participants once again listened to each piece of music used in this study (4 pieces with slow-tempo music sequences, 7 pieces with fast-tempo music sequences. See S1 Table) for 30 seconds, and were asked to rate their familiarity with each piece on a scale of 0 (never heard it before) to 15 (knows this piece very well) [29].

### Self-reported emotions when listening to music

The participants reported the arousal and valence after listening to music. For the assessment, we prepared a paper on which we had drawn two 15-cm lines as well as the music rating features. After the second saliva collection, the participants were asked to rate arousal (0-relaxing to 15-exciting) and valence (0-unpleasant to 15-pleasant). A significant difference was observed in arousal level (Wilcoxon signed-rank test, p = 0.00004, *r* = 0.80 for slow tempo vs fast tempo) and in valence level (Wilcoxon signed-rank test, p = 0.0077, *r* = 0.52 for slow tempo vs fast tempo) (S1 Fig). Although the valence for the fast-tempo music sequence was significantly lower than that for the slow-tempo music sequence, it indicated that the fast-tempo music sequence was sufficiently pleasant because the valence for the fast-tempo music sequence sufficiently exceeded the midpoint 7.5 (Wilcoxon signed-rank test, p = 0.0056, *r* = 0.54). The arousal for the fast-tempo music sequence was divided. In particular, the arousal rating for the fast-tempo music sequence was not significantly different from the midpoint despite some of the participants rating it 0~1 (very relaxing).

### Endocrinological measurements

We measured the oxytocin and cortisol levels in the participants’ saliva. Human saliva samples (3 mL/person) were directly collected in conical tubes from healthy men (age 21 to 34 years) between 14:00 and 17:00 h. Saliva was collected from participants via passive drool into a cold tube. The saliva was then divided into equal 1 mL amounts, two of which were used for the oxytocin assay and the other for the cortisol assay. Each sample for the oxytocin assay was immediately mixed with half the amount of protease inhibitor solution (0.5 mL) to prevent oxytocin degradation. Protease inhibitor cocktail solution was prepared immediately before use to avoid the hydrolysis of inhibitor peptides. All the samples including those used for the cortisol assay were frozen immediately and stored at -80°C until needed for the measurement.

#### Assay of oxytocin concentration

On the day of the oxytocin concentration measurement, a saliva sample was thawed and kept on ice. [^3^H]-labeled oxytocin was added to one group of tubes (“hot” samples), with which we evaluated the rate of oxytocin loss during processing until an enzyme-linked immunosorbent assay (ELISA) was performed. Another group of tubes (“cold” samples) was used in the oxytocin concentration measurement with ELISA. The radioactive counts of [3H]-labeled oxytocin added to the samples were adjusted to 20,000 using a liquid scintillation counter (Beckman Coulter, USA) with Clearsol (Nacalai Tesque, Japan) on each day of extraction processing.

Next, 1.5 mL of trifluoroacetic acid (TFA) (0.1% in double distilled water) was added to each saliva sample. After careful mixing, the samples were centrifuged at 2,000 rpm for 10 min at 4°C. The supernatant was carefully obtained for column purification. C18 columns (Waters, USA) were first washed with 5 mL of acetonitrile (ACN) and then with 12 mL of 0.1% TFA. Samples were applied to the column and were again washed with 12 mL of 0.1% TFA. Finally the columns were eluted with 6 mL of elution buffer composed of 0.1% TFA and ACN combined at a 40:60 ratio. The elution was collected and dried under a constant airflow in a chemical fume hood at 4°C. After drying, 200 μl of the assay buffer from the ELISA kit was added to each sample thus concentrating the samples (200 μl from 1 ml saliva), resulting in a sample with a salivary concentration five times higher than the original saliva. The counts in the hot samples were measured in a liquid scintillation counter and recorded for the recovery rate calculation. Cold samples were employed for oxytocin ELISA.

Oxytocin ELISA was carried out according to the manufacturer’s instructions with minor modifications. The absorption at 405 nm was read with a Model 550 plate reader (BioRad, UK). A standard logarithmic curve was generated from 8 doses of oxytocin standard (1000, 500, 250, 125, 62.5, 31.3, 15.7, 7.81 pg/mL) whose coefficient of correlation *r*^*2*^ was around 0.98. The standard curves were similar across all the assays.

When the oxytocin concentration of the sample was calculated as “C” pg/mL, the true oxytocin concentration “Ct” was evaluated as follows:

Ct=C*1/5*20000/a(pg/mL), (1)

where “a” indicates the radioactivity count of the corresponding hot sample.

#### Assay of cortisol concentration

Saliva samples (1 mL) to be used for cortisol measurement were stored at -80°C. The cortisol concentration in saliva was determined using a liquid chromatography-tandem mass spectrometry (LC-MS/MS) system. All the analyzes of the cortisol level in saliva were performed using the standard protocols by ASKA Pharma Medical Co. Ltd. (Japan) [30,31], which has significant experience as regards various types of steroid hormonal assay. Staff at the company were not informed of the sample content or the nature of the study.

### Physiological measurements

ECGs were used to measure interbeat intervals (R-R intervals). Analogue data were amplified and digitized with a BIOPAC MP150 (BIOPAC Systems, USA). The sampling rate was 1,250 Hz.

#### HR and heart rate variability (HRV)

To calculate the R-R intervals in the ECG measurement, R-wave detection was performed with AcqKnowledge (analysis software produced by BIOPAC MP150, USA), and the result was visually screened to eliminate any inappropriate R-wave detection related to artifacts such as movement. The appropriately collected R-R interval data were resampled at 10 Hz by cubic spline interpolation. For HR analysis, the interpolated R-R interval data were converted to second-by-second values and expressed in beats per minute (bpm) by dividing 60 by each value. For HRV analysis, a fast Fourier transformation (FFT) was applied to this interpolated R-R interval data after removing the linear trend to calculate the HRV power spectra using a Hanning window. Low frequency (LF) and high frequency (HF) components were obtained by integrating the power spectra over their respective ranges of 0.04–0.15 Hz and 0.15–0.40 Hz. The magnitude of the HF and the ratio of LF to HF (LF/HF) correspond to the strength of the vagal activity [32] and the sympathovagal balance [33], respectively. The magnitude of the LF involves both vagal and sympathetic nerve activity [34]. FFT was applied to each 2-min window of the interpolated data series of R-R intervals. The magnitude of each spectral component was evaluated by using the natural logarithms of the power (lnLF and lnHF). The ratio of the LF component to the HF component (LF/HF ratio) was evaluated by dividing lnLF by lnHF (lnLF/lnHF).

### Design and experimental procedure

We used a 2 x 2 within-subjects design, where Time (before and after music stimuli) and Tempo (slow and fast tempi) were independent variables. This study dealt with the following dependent variables; the salivary oxytocin and cortisol levels, and cardiovascular responses (lnLF, lnHF, lnLF/lnHF, and HR), all of which are described above.

This study consisted of two listening sessions a day, an earlier listening session (14:30–15:30) and a later listening session (16:00–17:00). Participants came to the laboratory for 2 days, and on each day they listened to one of the experimental music sequences (slow- or fast-tempo music sequence). On each experiment day, 2 participants came to our laboratory and one of them was assigned to the earlier listening session and the other to the later listening session. On the second day, they were assigned to the same listening session time. The order of the music sequences, the order of the pieces of music in each sequence and the assignment to the earlier or later listening session were randomized.

The participants were instructed not to drink anything containing alcohol or caffeine from 20:00 on the day before their participation, and not to consume anything except still water after lunch (12:00–13:00) on the day of the experiment. On the day of the experiment, they were given general information about the experiment on arrival and their written consent was obtained. The experimental procedure consisted of four periods: rest period → saliva collection period 1→ music period → saliva collection period 2. Prior to the listening session, the participants sat on a sofa, and were attached to ECG transducer electrodes for 10 min to familiarize them with the experimental environment. This time period is referred to as the “rest period”. The last 2-min of the ECG recording was regarded as the baseline. We then collected saliva samples of over 3 mL from the participants via passive drool into a cold tube for hormone measurements (saliva collection period 1). This saliva sample was used as the baseline for the salivary levels of oxytocin and cortisol. When the listening session started (music period), the participants were presented with one of the experimental music sequences, namely a slow- or fast-tempo music sequence with a maximum level of 75 dB SPL (A), for 20 min. There was then a second period during which a saliva sample was obtained (saliva collection period 2). After the second collection of saliva, the participants were asked to rate arousal and valence. After all the procedures (after rating arousal and valence on the second day), they were asked to report their familiarity with pieces of music that they heard in this study. Before reporting their familiarity, they once again listened to each piece for 30 seconds.

**Fig 1:**
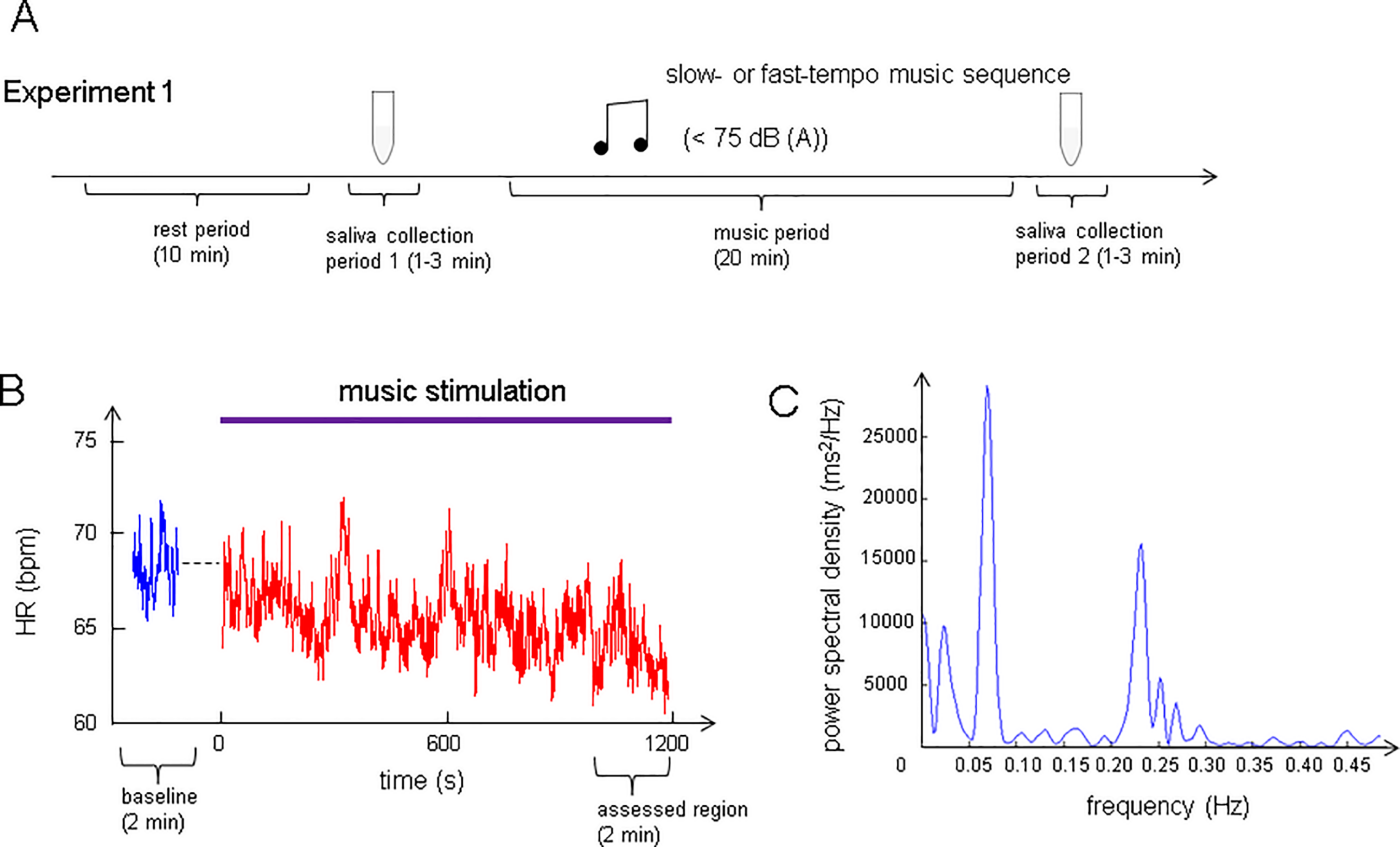
Experimental procedure and analysis of HR and HRV. (A) Paradigm of Experiment 1. The participants experienced one 10-min silent period as a rest period, two saliva collection periods (1 and 2), each of which was 1–3 min long, and one 20-min music period. After the rest period, in saliva collection period 1 we took a saliva sample of over 3 mL from the participants for hormone assays. In the next music period, which lasted 20 min, a slow- or fast-tempo music sequence was presented. Finally, the participants again provided saliva during saliva collection period 2. The participants heard one of the slow- and fast-tempo music sequences on each day. The maximum sound pressure level of the music was regulated at 75 dB (A) in the slow mode. (B) A sample recording of the HR during baseline recording and music stimulation for 20 min. The HR was calculated with the following equation: HR (bpm) = 60*1000/R-R interval (ms). t = 0 indicates the start of the music. To evaluate the effect of music listening on the autonomic nerves, we analyzed the HR and HRV data in the t = 1080 to 1200 region (assessed region) and compared them with that at the baseline. (C) A sample analysis of HRV. Low-frequency (LF) and high-frequency (HF) components were obtained by integrating the power spectra over their respective ranges of 0.04–0.15 Hz and 0.15–0.40 Hz.

The experimental procedure is summarized in Fig 1A. A sample time series of the HR and a sample analysis of the HRV are shown in Fig 1B and 1C, respectively.

### Data analysis

Data are presented as means ± SEM, and the probability value p < 0 .05 was considered to be statistically significant. We analyzed the influence of listening to music on changes in the secretion of oxytocin and cortisol into saliva with a two-factor repeated measures ANOVA with Tempo (slow and fast) and Time (before and after music stimuli) as factors. The influence on arousal and valence levels was analyzed with a Wilcoxon signed-rank test. The same statistical procedure was applied to the HR and HRV data as was applied to the hormone level data noted above. We used Pearson’s correlation method to examine the statistical correlation between the ratio of the changes in oxytocin and cortisol levels and that in each parameter of the autonomic responses. We used Spearman’s correlation method to examine the statistical correlation between the ratio of the change in the oxytocin or cortisol level and the arousal or valence level.

Huynh-Feldt corrections were applied where appropriate.

## Results

### Self-reported familiarity with music pieces

The participants rated their familiarity with a total of 11 pieces of music, consisting of 4 pieces of slow-tempo music and 7 pieces of fast-tempo music, all of which were used in the experiment. All of the participants rated every music piece at 0, thus indicating that none of them had heard any of the Chopin pieces used in the experiment.

### Salivary oxytocin and cortisol levels before and after listening to slow- and fast-tempo music sequences

We performed a two-factor repeated measures ANOVA on the oxytocin and cortisol levels, with Tempo (slow and fast) and Time (before and after music stimuli) as within-subjects factors. The data obtained for 3 participants were excluded from the oxytocin analysis because there was an abnormal result as regards the oxytocin concentration in their saliva (it exceeded 700 pg/ml). The data obtained for 1 participant were excluded from the cortisol analysis because there was insufficient saliva for the cortisol analysis. Consequently, we analyzed data for 23 participants for the oxytocin analysis, and 25 participants for the cortisol analysis.

**Fig 2:**
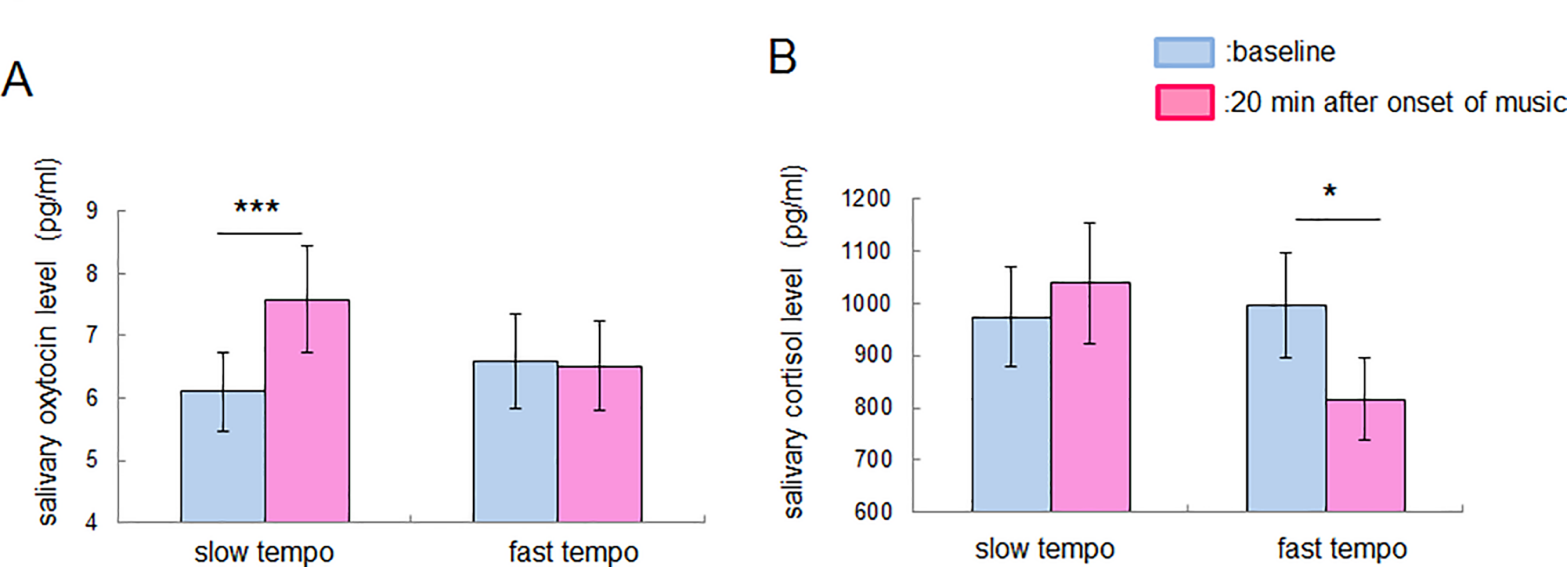
Effect of music stimuli on salivary oxytocin and cortisol levels. (A) Salivary oxytocin level before and after 20 min of music stimulation. The salivary oxytocin level was greater after listening to the slow-tempo music sequence. (B) Salivary cortisol level before and after 20 min of music stimulation. The salivary cortisol level was lower after listening to the fast-tempo music sequence. Data are presented as means ± SEM; * p < 0.05, *** p < 0.001 for a simple main effect test.

The ANOVA revealed a significant Tempo x Time interaction for the oxytocin level (F (1,22) = 13.44, p = 0.0014, partial *η*^*2*^ = 0.38, n = 23 participants, no significant main effect) and that for the cortisol level (F (1,24) = 8.31, p = 0.0082, partial *η*^*2*^ = 0.26, n = 25 participants, no significant main effect) (Fig 2A and 2B). A simple main effect test demonstrated that the salivary oxytocin level was significantly greater (F (1,44) = 13.64, p = 0.0007, n = 23 participants) than the baseline after the participants listened to the slow-tempo music sequence, while no significant change in the salivary cortisol level was observed (F (1,48) = 0.72, p = 0.40, n = 25 participants). The salivary cortisol level was significantly lower (F (1,48) = 5.65, p = 0.022, n = 25 participants) than the baseline after the participants listened to the fast-tempo music sequence, while no significant change in the salivary oxytocin level was observed (F (1,44) = 0.032, p = 0.86, n = 23 participants).

### Effect of listening to slow- and fast-tempo music sequences on autonomic nerve activity

**Fig 3:**
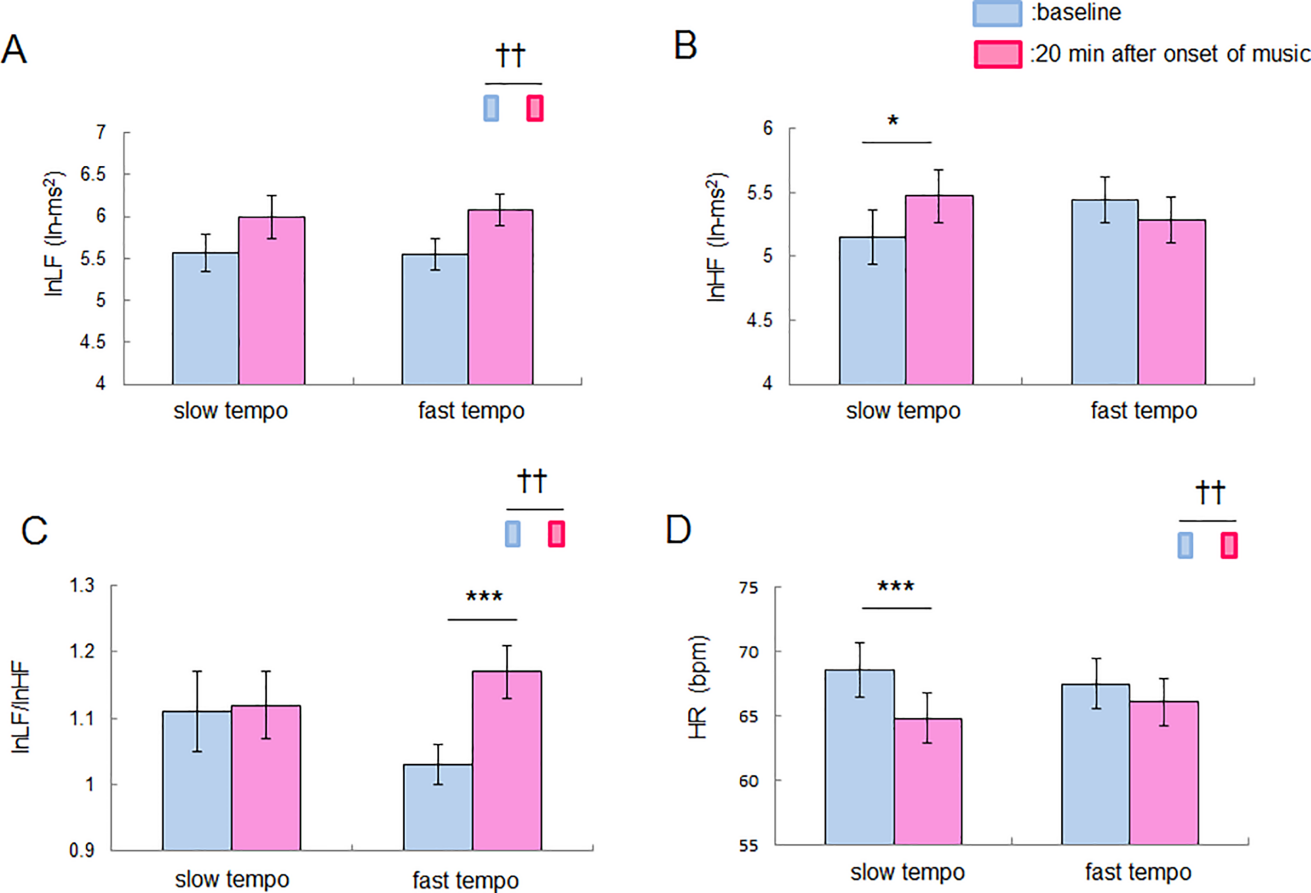
Effect of music stimuli on indices of autonomic nerve activity. (A) LnLF before and after music stimulation for 20 min. The main effect of Time was observed. (B) LnHF before and after music stimulation for 20 min. LnHF increased significantly after the participants listened to a slow-tempo music sequence. (C) LnLF/lnHF before and after music stimulation for 20 min. LnLF/lnHF increased significantly after the participants listened to a fast-tempo music sequence. (D) HR before and after music stimulation for 20 min. HR decreased significantly after the participants listened to a slow-tempo music sequence. Data are presented as means ± SEM; * p < 0.05, *** p < 0.001 for a simple main effect test; †† p < 0.01 for a main effect test.

A two-factor repeated measures ANOVA with Tempo (slow and fast) and Time (before and after music stimuli) as within-subjects factors revealed a significant main effect of Time (F (1,25) = 12.20, p = 0.0018, partial *η*^*2*^ = 0.33, n = 26 participants, no significant interaction effect) for lnLF, a significant Tempo x Time interaction (F (1,25) = 6.91, p = 0.015, partial *η*^*2*^ = 0.22, n = 26 participants, no significant main effect) for lnHF, a significant Tempo x Time interaction (F (1,25) = 4.42, p = 0.046, partial *η*^*2*^ = 0.15, n = 26 participants) and a main effect of Time (F (1,25) = 11.75, p = 0.0021, partial *η*^*2*^ = 0.32, n = 26 participants) for lnLF/lnHF, and a significant Tempo x Time interaction (F (1,25) = 7.29, p = 0.012, partial *η*^*2*^ = 0.23, n = 26 participants) and a main effect of Time (F (1,25) = 9.41, p = 0.0051, partial *η*^*2*^ = 0.27, n = 26 participants) for HR (Fig 3A–3D). A simple main effect test demonstrated that lnHF was significantly greater (F (1,50) = 5.53, p = 0.023, n = 26 participants) and the HR was lower (F (1,50) = 15.66, p = 0.0003, n = 26 participants) than the baseline after listening to a slow-tempo music sequence, while there was no significant change in lnLF/lnHF (F (1,50) = 0.017, p = 0.90, n = 26 participants). LnLF/lnHF increased significantly (F (1,50) = 13.26, p = 0.0007, n = 26 participants) after the participants listened to a fast-tempo music sequence compared with the baseline, while there was no significant change in lnHF (F (1,50) = 1.51, p = 0.22, n = 26 participants) or HR (F (1,50) = 2.29, p = 0.14, n = 26 participants).

### Correlation of changes in salivary oxytocin and cortisol levels with changes in autonomic nerve activity and self-reported emotion

To investigate the possibility that oxytocin and cortisol are chemical bases of excitation and relaxation induced by music listening, we analyzed the correlation of the changes in oxytocin and cortisol levels with the changes in autonomic nerve activity and self-reported emotion.

**Fig 4:**
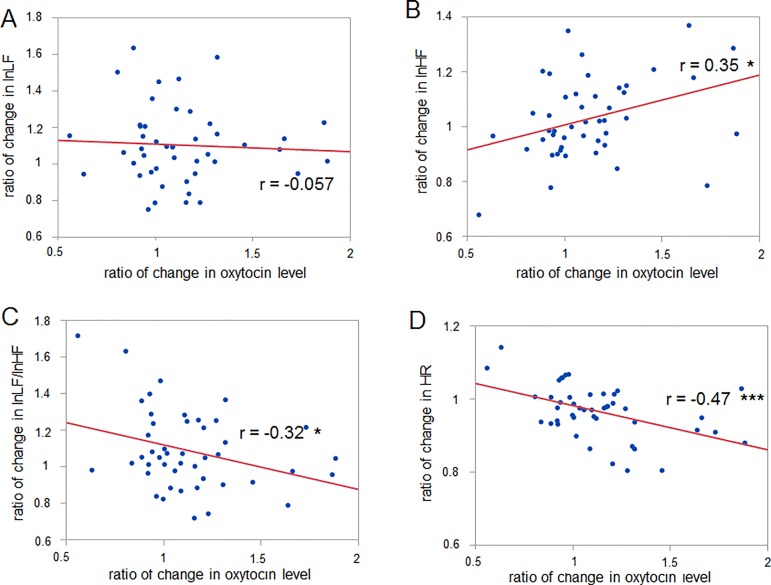
Correlation between the change in the oxytocin level and changes in autonomic nerve activity. (A-D) The change in the oxytocin level shows a significantly positive correlation with changes in (B) lnHF and a negative correlation with those in (C) lnLF/lnHF and (D) HR, while no significant correlation is observed between the change in oxytocin level and that in (A) lnLF. * p < 0.05, *** p < 0.001 (Pearson’s correlation method). Pearson’s correlation coefficient r is described.

**Fig 5:**
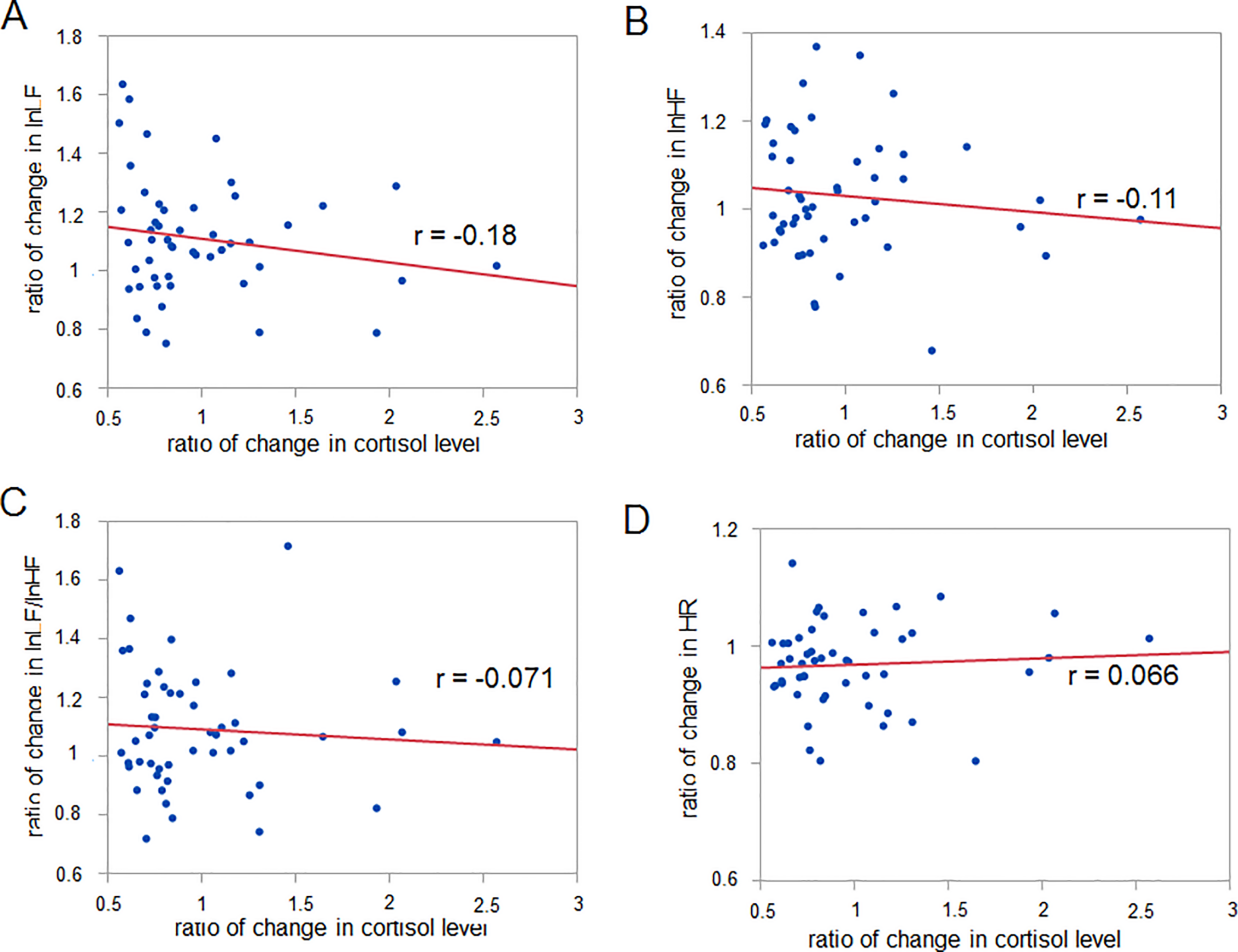
Correlation between change in cortisol level and changes in autonomic nerve activity. (A-D) The change in the cortisol level shows no significant correlation with changes in autonomic nerve activity (A-D). Pearson’s correlation coefficient r is described.

Pearson’s method was used to analyze the correlation between the changes in oxytocin and cortisol levels and in autonomic nerve activity. The results showed that the ratio of the increase in the oxytocin level correlated significantly with that of the increase in lnHF, that of the decrease in lnLF/lnHF, and that of the decrease in HR (r = 0.35, p = 0.017 for oxytocin vs lnHF; r = -0.32, p = 0.031 for oxytocin vs lnLF/lnHF, r = -0.47, p = 0.0009 for oxytocin vs HR, 46 data points) (Fig 4B–4D), and was uncorrelated with the change in lnLF (r = -0.057, p = 0.71 for oxytocin vs lnLF, 46 data points) (Fig 4A). On the other hand, there was no significant correlation between the change in the cortisol level and that in autonomic nerve activity (r = -0.18, p = 0.22 for cortisol vs lnLF; r = -0.11, p = 0.44 for cortisol vs lnHF; r = -0.071, p = 0.62 for cortisol vs lnLF/lnHF; r = 0.066, p = 0.65 for cortisol vs HR, 50 data points) (Fig 5A–5D).

**Fig 6:**
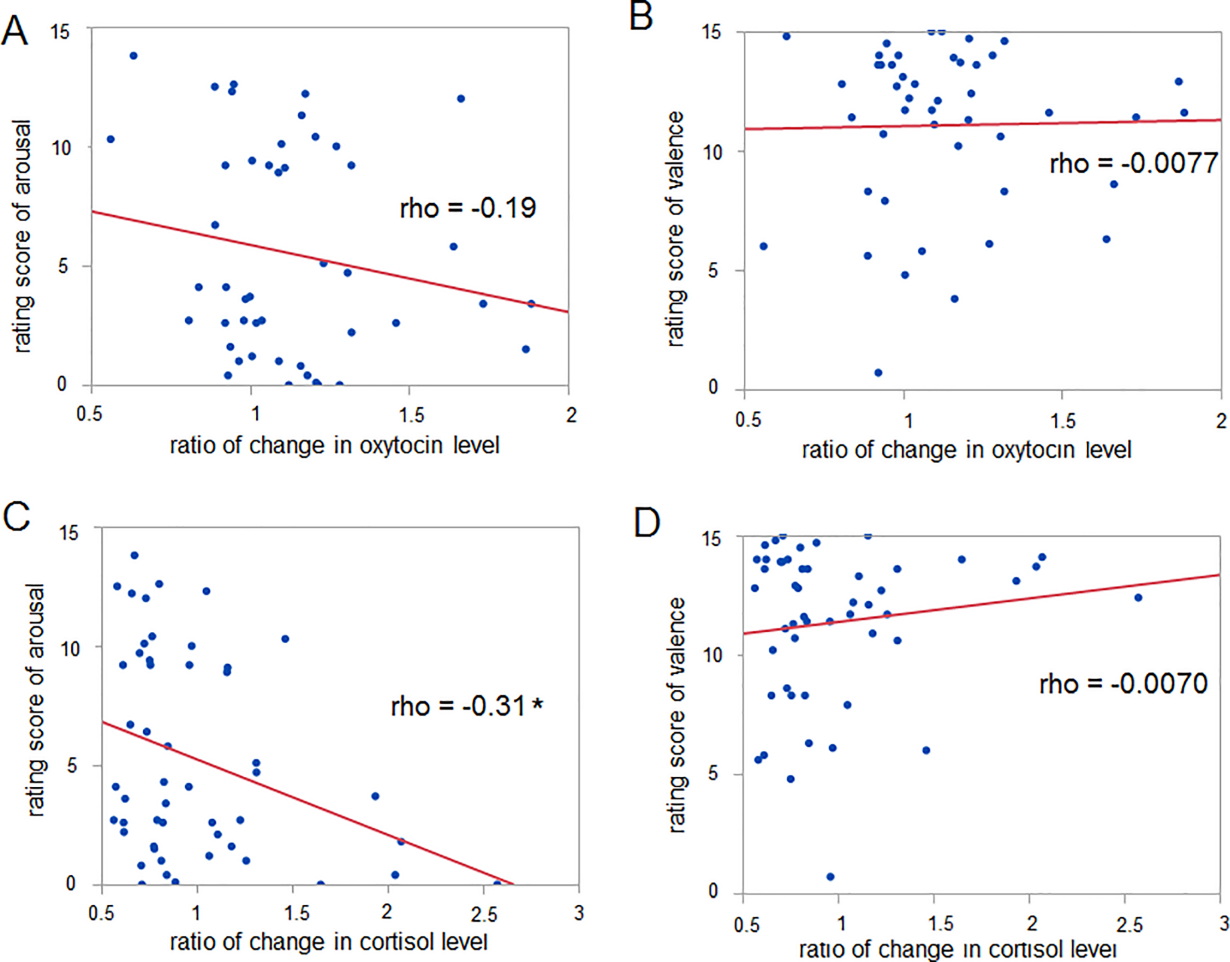
Correlation between the change in the oxytocin or cortisol level and self-reported emotions. (A) Change in the oxytocin level vs arousal, (B) change in the oxytocin level vs valence, (C) change in the cortisol level vs arousal, and (D) change in the cortisol level vs valence. The change in the cortisol level vs arousal alone shows a significant correlation. * p < 0.05 with Spearman’s correlation method. Spearman’s correlation coefficient rho is described.

Spearman’s method was used to analyze the correlation between the changes in oxytocin and cortisol levels and self-reported emotions. The results showed that the ratio of the change in the oxytocin level did not show any significant correlation with arousal level (rho = -0.19, p = 0.20, 46 data points) or valence level (rho = -0.0077, p = 0.96, 46 data points) (Fig 6A and 6B). On the other hand, the ratio of the change in the cortisol level showed a significant negative correlation with arousal level (rho = -0.31, p = 0.031, 50 data points) but did not show any significant correlation with valence level (rho = -0.0070, p = 0.96, 50 data points) (Fig 6C and 6D).

## Discussion

Listening to music can have a range of effects on human beings including inducing relaxation [7–9] and excitation [3–6]. Although it is well known that the tempo of music is a critical factor in determining whether the effect of listening to music is exciting [15] or relaxing [16], it has been unclear what chemical bases underlie these relaxation and excitation effects. To address the question, we assume oxytocin and cortisol to be candidates for the chemical bases of relaxation and excitation, respectively. Oxytocin has anti-stress [18], anti-anxiety effects [19] and has the potential to facilitate vagal activity [20]. Corticosterone, a glucocorticoid of rodents, increases the firing rate of cardiovascular neurons in the RVLM in rats [23]. Taken together, we therefore hypothesized that listening to relaxing music with a slow tempo would increase the oxytocin level, while listening to exciting music with a fast tempo would increase the cortisol level. This study found that the salivary oxytocin level and lnHF, which is an index of vagal activity, increased when a slow-tempo music sequence was presented, while the salivary cortisol level decreased and lnLF/lnHF, which is an index of the sympathovagal balance, increased when a fast-tempo music sequence was presented. Intriguingly, the increase in the oxytocin level correlated significantly with the increase in lnHF, the decrease in lnLF/lnHF and the decrease in HR. This indicates that oxytocin is related to the dominance of the parasympathetic nerve activity. On the other hand, the decrease in the cortisol level correlated significantly with the arousal level. Therefore, we infer that listening to slow/ fast-tempo music induces changes in oxytocin/cortisol levels, which are involved in physiological relaxation and emotional excitation, respectively.

The slow-tempo music sequence used in the current study can be regarded as soothing music because the self-reported arousal level was sufficiently small and the valence level was sufficiently large (S1 Fig). Our results, which show a significant increase in the salivary oxytocin and lnHF and a significant reduction in the HR induced by listening to the slow-tempo music sequence, are consistent with an earlier study demonstrating that soothing music listening enhances the plasma oxytocin level [35], reduces the HR [36], and increases the amplitude of respiratory sinus arrhythmia (RSA) (equal to the HF component of HRV) [37]. When we consider another study demonstrating that the application of oxytocin protects against the social stress-induced suppression of RSA [20], we can assume that oxytocin secretion induced by music listening is related to vagal nerve activity originating from the nucleus ambiguous (NA) [32]. Our results demonstrated that the change in the oxytocin level accompanied by music listening correlates positively with that in lnHF (Fig 4B). It should be noted, however, that there is no significant immunostaining of the oxytocin receptors in NA, whereas there is in the nucleus tractus solitarius (NTS) [38]. Oxytocin possibly has an indirect influence on the enhancement of NA-derived vagal activity through NTS. However, we could not find any correlation between the music listening-related change in the oxytocin level and self-reported relaxation, suggesting that oxytocin is involved not in emotional relaxation but in physiological relaxation. This is consistent with an earlier study demonstrating that oxytocin is related to implicit empathic responses rather than self-reported empathy scores [39].

By measuring the cortisol level and autonomic nerve activity, previous studies have indicated that listening to music can reduce mental stress or physiological dysfunction. Listening to relaxing music can suppress the stress-induced elevation of the cortisol level [7,8], while one study reported that listening to relaxing music fails to induce such effects [9]. Our results appear to be inconsistent with both earlier studies and our hypothesis because we observed that listening to a fast- rather than a slow-tempo music sequence resulted in a decrease rather than an increase in the cortisol level (Fig 2B). This inconsistency might derive from the individual variability of the glucocorticoid responses [40] or the complex relationship between music components, emotional state and relaxation [41,42]. Another reason could be the complexity of the sympathetic nerve activity. Our hypothesis that listening to a fast-tempo music sequence induces an increase in the cortisol level is based on an earlier study demonstrating that the introduction of corticosterone into the RVLM increases the firing rate of cardiovascular neurons in the RVLM [23]. However, there is another study, indicating that glucocorticoid agonist reduces the sympathetic outflow [43]. That would be why the change in the cortisol level resulting from listening to music had no correlation with changes in autonomic nerve activity (Fig 5A–5D). Such dissociation between autonomic nerve activity and the change in the cortisol level was reported in earlier studies [44,45]. Interestingly, we found that that the cortisol level is related to the self-reported arousal score (Fig 6C). In contrast to the case with oxytocin, cortisol is involved in emotional excitation. Our result showing a negative correlation between the music listening-related change in the cortisol level and the self-reported arousal score is consistent with earlier studies in the sense of there being a negative relationship between cortisol and emotional arousal [46,47].

In conclusion, we found that the salivary level of oxytocin increased when a slow-tempo music sequence was presented, while the salivary level of cortisol decreased when a fast-tempo music sequence was presented. Since the change in the salivary oxytocin level was correlated with the change in the parasympathetic nerve activity and the change in the salivary cortisol level was correlated with the arousal level, we suggest that each hormonal response is involved in the relaxation and excitation in a different way when listening to music.

## Limitations

Since we wanted to examine the effect of naturally audible and comfortable music, we selected professionally played pieces that were taken from commercially available CDs. To prepare slow-tempo relaxing and fast-tempo exciting music sequences, we selected 4 slow tempo and 7 fast tempo piano pieces, all of which were composed by Chopin. To confirm that the difference between the subjective impressions of the two music classes is predominantly characterized in terms of tempo, we asked six music experts to rate the following music features; tempo (0-slow to 15-fast), rhythm (0-vague to 15-outstanding), pitch level (0-low to 15-high), pitch range (0-narrow to 15-wide), harmonic complexity (0-simple to 15-complex), and consonance (0-dissonant to 15-consonant) [27]. We observed a significant difference between the classes only in tempo (S2 Table).

It is, however, premature to conclude that musical tempo is the essential factor behind the physiological responses observed in the present study. First, the present experimental design, which involved adopting human subjects, was not sufficient to probe the causality between music listening and the physiological responses. Also, it should be remembered that a subjective tempo is likely determined by complex interactions between various acoustical and detailed musical features, and that the way of integrating those features to derive tempo perception and cognition can vary among individuals. Thus, there is a possibility that other unknown features, rather than the resulting tempo, might be the essential factor. Interestingly, however, with this scheme we found a significant correlation between the change in salivary oxytocin level and the change in autonomic responses (lnHF, lnLF/lnHF, and HR), and also between the changes in the salivary cortisol level and arousal level. A future direction will involve examining the effects of different composers and controlling the music features to uncover the mechanisms by which endocrinological systems are regulated when listening to music.

In this study, none of the participants were in the habit of listening to classic music or piano pieces. None of them knew any of the pieces by Chopin used in this study. These participant characteristics must be reflected in the results of arousal and valence for the Chopin compositions used in this study.

## Supporting information

S1 Fig The effect of 20 min of music stimulation on arousal and valence levels. (PPTX)

S1 Table Characteristics of the music pieces used in the experiment. (DOCX)

S2 Table Comparison of the six features of the music pieces used in the experiment for slow- and fast-tempo music sequences. (DOCX)

S1 Text Legends of S1 Fig, S1 Table and S2 Table. (DOCX)

S1 File Data set 1. This includes all the original data from which the results shown in Figs 1–6 were produced.
(XLS)

S2 File Data set 2. This includes all the rating scores for music pieces assessed by 6 music experts.
(XLSX)
