# Supplementary material for: Increase in salivary oxytocin and decrease in salivary cortisol after listening to relaxing slow-tempo and exciting fast-tempo music
Source: PLoS One. 2017 Dec 6;12(12):e0189075. doi: 10.1371/journal.pone.0189075 (PMC5718605; doi:10.1371/journal.pone.0189075)
Supplement: S1 Fig — (PPTX) [file pone.0189075.s001.pptx]

## Slide 1
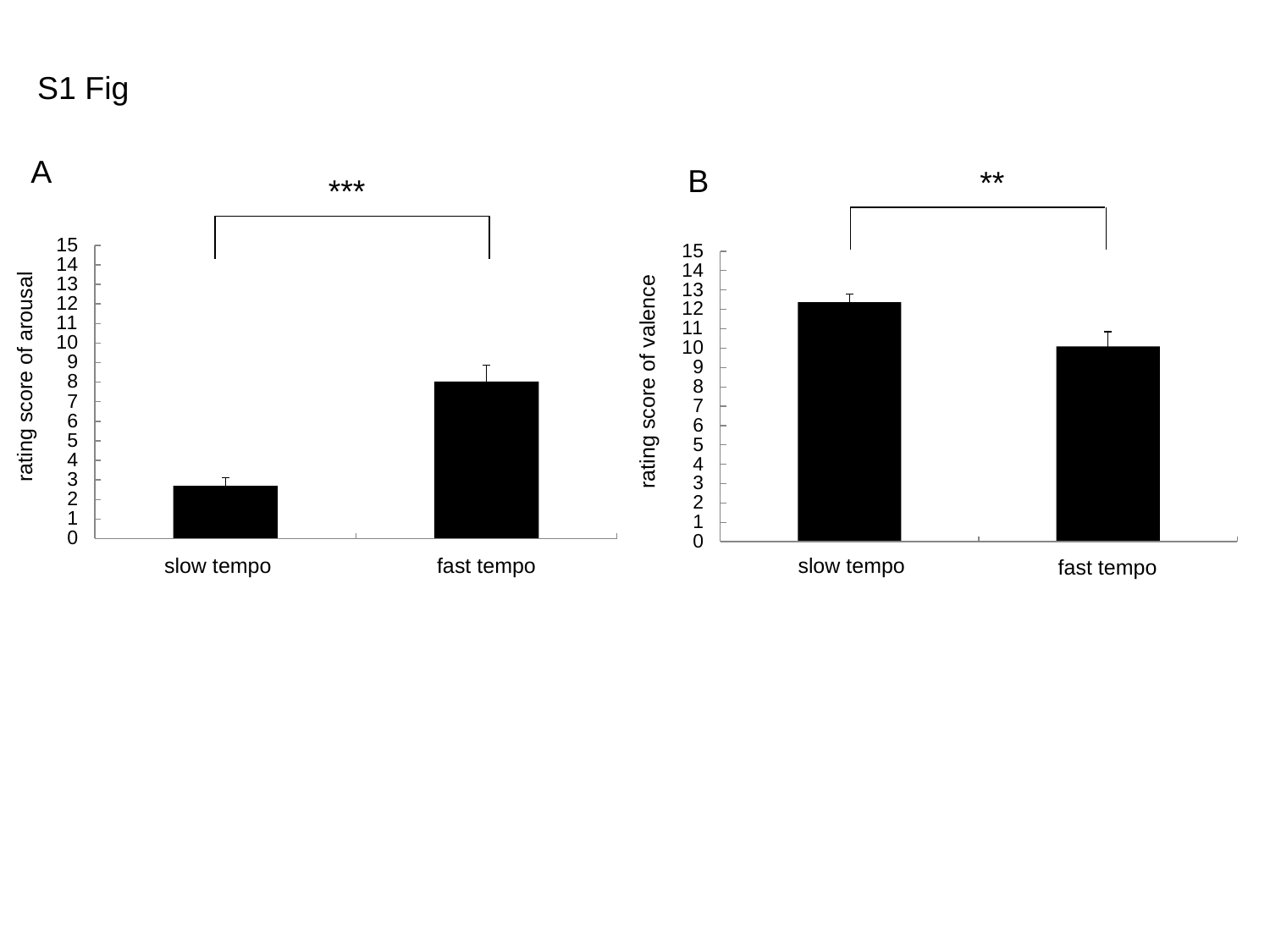

S1 Fig
A
B
**
***
rating score of arousal
rating score of valence
slow tempo
fast tempo
slow tempo
fast tempo
