# Supplementary material for: Increase in salivary oxytocin and decrease in salivary cortisol after listening to relaxing slow-tempo and exciting fast-tempo music
Source: PLoS One. 2017 Dec 6;12(12):e0189075. doi: 10.1371/journal.pone.0189075 (PMC5718605; doi:10.1371/journal.pone.0189075)
Supplement: S1 Table — (DOCX) [file pone.0189075.s002.docx]

---------Rating score by music experts-----------

| type | tune name | opus number | tempo (touches/min) | tempo  (crotchets/min) | tempo | rhythm | pitch level | pitch range | harmonic  complexity | consonance |
| --- | --- | --- | --- | --- | --- | --- | --- | --- | --- | --- |
| slow tempo | Berceuse | Op. 57 | 213.8 | 50.0 | 3.6±0.8 | 8.3±0.9 | 8.6±0.6 | 9.3±0.8 | 7.9±0.8 | 10.0±0.9 |
|  | Nocturne No. 8 | Op. 27-2 | 117.3 | 42.3 | 4.1±0.6 | 9.1±0.7 | 7.5±0.7 | 7.6±0.6 | 8.9±0.8 | 8.8±1.2 |
|  | Nocturne No. 18 | Op. 62-2 | 125.1 | 62.5 | 4.6±0.9 | 9.3±0.9 | 7.2±0.6 | 8.8±0.9 | 8.6±0.4 | 8.1±0.5 |
|  | Andante spianato | Op. 22 | 138.0 | 68.9 | 4.7±0.7 | 8.5±0.9 | 10.1±0.5 | 8.5±0.6 | 6.2±0.5 | 9.9±1.2 |
| fast tempo | Etude No. 1 | Op. 10-1 | 622.5 | 170.8 | 11.4±1.2 | 8.4±1.5 | 9.1±0.9 | 10.7±0.9 | 6.3±0.9 | 9.2±1.1 |
|  | Etude No. 5 | Op. 10-5 | 572.4 | 106.3 | 10.3±0.6 | 7.8±1.4 | 10.3±0.8 | 9.3±1.2 | 6.2±1.1 | 9.8±0.5 |
|  | Waltz No. 1 | Op. 18 | 294.6 | 210.5 | 8.7±0.3 | 10.0±0.7 | 7.8±0.5 | 9.3±0.7 | 8.3±0.5 | 9.1±1.1 |
|  | Waltz No. 4 | Op. 34-3 | 362.5 | 268.7 | 12.1±0.8 | 9.0±0.8 | 9.3±0.5 | 9.2±0.9 | 7.2±0.9 | 9.9±0.7 |
|  | Waltz No. 5 | Op. 42 | 322.8 | 202.4 | 9.2±0.4 | 10.5±0.7 | 8.0±0.7 | 9.3±0.7 | 8.9±0.7 | 8.7±0.5 |
|  | Waltz No. 6 | Op. 64-1 | 362.5 | 215.4 | 10.3±0.7 | 10.0±0.7 | 9.7±0.9 | 8.8±0.5 | 6.2±0.9 | 10.9±0.8 |
|  | Mazurka No. 23 | Op. 33-2 | 384.5 | 225.4 | 8.9±1.0 | 8.2±1.2 | 7.2±0.6 | 7.4±0.3 | 6.5±0.9 | 9.0±0.7 |
