# Supplementary material for: Increase in salivary oxytocin and decrease in salivary cortisol after listening to relaxing slow-tempo and exciting fast-tempo music
Source: PLoS One. 2017 Dec 6;12(12):e0189075. doi: 10.1371/journal.pone.0189075 (PMC5718605; doi:10.1371/journal.pone.0189075)
Supplement: S2 Table — (DOCX) [file pone.0189075.s003.docx]

|  | slow tempo | fast tempo | t value | p value |
| --- | --- | --- | --- | --- |
| tempo | 4.3±0.5 | 10.1±0.5 | 9.89 | 0.0002 S |
| rhythm | 8.8±0.7 | 9.2±0.8 | 0.26 | 0.81 NS |
| pitch level | 8.4±0.4 | 8.7±0.5 | 0.71 | 0.51 NS |
| pitch range | 8.6±0.3 | 9.2±0.3 | 1.74 | 0.14 NS |
| harmonic complexity | 7.9±0.4 | 7.1±0.5 | 1.43 | 0.21 NS |
| consonance | 9.2±0.7 | 9.5±0.5 | 0.50 | 0.64 NS |
