# Supplementary material for: Increase in salivary oxytocin and decrease in salivary cortisol after listening to relaxing slow-tempo and exciting fast-tempo music
Source: PLoS One. 2017 Dec 6;12(12):e0189075. doi: 10.1371/journal.pone.0189075 (PMC5718605; doi:10.1371/journal.pone.0189075)
Supplement: S1 Text — (DOCX) [file pone.0189075.s004.docx]

**Supporting Information**

Increase in salivary oxytocin and decrease in salivary cortisol after listening to relaxing slow-tempo and exciting fast-tempo music

Yuuki Ooishi, Hideo Mukai, Ken Watanabe, Suguru Kawato, and Makio Kashino

**Legends**

**S1 Fig. The effect of 20 min of music stimulation on arousal and valence levels (n = 26 participants).** (A) Arousal level for the slow-tempo music sequence is lower than that for the fast-tempo music sequence. (B) Valence level for the slow-tempo music sequence is higher than that for the fast-tempo music sequence. Data are presented as means ± SEM; ** p < 0.01, *** p < 0.001 for Wilcoxon signed-rank test.

**S1 Table. Characteristics of the music pieces used in the experiment.** Slow- and fast-tempo music sequences consisted of 4 and 7 Chopin piano pieces, respectively. Each sequence lasted 20 min. The tempo was measured in touches/min and crotchets/min, where 1 touch means striking 1 piano note or chord with the right hand. The six arrays on the right are the scores of the music features (mean ± SEM) rated by six music experts; tempo (0-slow to 15-fast), rhythm (0-vague to 15-outstanding), pitch level (0-low to 15-high), pitch range (0-narrow to 15-wide), harmonic complexity (0-simple to 15-complex), and consonance (0-dissonant to 15-consonant).

**S2 Table. Comparison of the six features of the music pieces used in the experiment for slow- and fast- tempo music sequences.** For each feature, the data obtained from one music expert were averaged within slow- (4 pieces) and fast-tempo (7 pieces) music sequences, respectively. The average data for each music expert were used to perform a statistical analysis by using a paired t-test for slow- and fast-tempo music sequences. The t value and the p value are the results of this analysis. NS: no significance, S: significance.
